# Estimating insect pest density using the physiological index of crop leaf

**DOI:** 10.3389/fpls.2023.1152698

**Published:** 2023-08-10

**Authors:** Meng Chen, Xiang-Dong Liu

**Affiliations:** Department of Entomology, Nanjing Agricultural University, Nanjing, China

**Keywords:** density estimation, *Nilaparvata lugens*, rice leaf, silicon, soluble sugar, SPAD, water content

## Abstract

Estimating population density is a fundamental study in ecology and crop pest management. The density estimation of small-scale animals, such as insects, is a challenging task due to the large quantity and low visibility. An herbivorous insect is the big enemy of crops, which often causes serious losses. Feeding of insects results in changes in physiology-related chemical compositions of crops, but it is unknown whether these changes can be used to estimate the population density of pests. The brown planthopper (BPH), *Nilaparvata lugens*, is a serious insect pest hiding under rice canopy to suck the sap of rice stems. BPH density is a crucial indicator for determining whether the control using pesticides will be carried out or not. Estimating BPH density is still dependent on manmade survey and light-trap methods, which are time-consuming and low-efficient. Here, we developed a new method based on the physiological traits of rice leaves. The feeding of BPHs significantly decreased the contents of chlorophyll (the SPAD readings), water, silicon, and soluble sugar in rice leaves. Four ratio physiological indices based on these four physiological traits of the BPH-damaged rice leaves to those of healthy leaves were established, and they were significantly correlated with BPH density in rice plants. A rice growth stage-independent linear model based on the four ratio physiological indices and adding the other two variables, BPH damage duration and population increase rate, was developed. This model exhibited a reasonable accuracy for estimating BPH density. This new method will promote the development of density estimation of pest populations toward nonprofessionalization and automation.

## Introduction

Density estimation of animals is an important and challenging task in ecology and crop pest management that requires multiple skills, including sampling technique, species recognition and counting, data analysis, and density computing. The traditional methods for density estimation are mainly dependent on manmade investigation and traps using specific devices, such as light and net (Anderson et al., 1983; Parmenter and Macmahon, 1989; Keeping, 2014), although automatic recording via camera or other devices has been developed (Silveira et al., 2003; Rowcliffe et al., 2008; Nakashima et al., 2018; Yajima and Nakashima, 2021). These traditional methods totally rely on manual labor, and they are time-consuming and low-efficient. So, simple, less labor-intensive, and highly efficient methods are necessary in ecology and plant protection.

The brown planthopper (BPH), *Nilaparvata lugens*, is a devastating small-body insect pest of rice throughout Asia that hides under the rice canopy and sucks sap from the stem, often causing heavy losses of rice yield (Dyck and Thomas, 1979; Sogawa, 1982; Heong et al., 2015). Due to the small body size, large population density, and complex rice paddy system, estimating the density of BPH is a professional and highly labor-intensive task. Monitoring and prewarning of BPH population dynamics are fundamental to effectively managing this pest and mitigating the loss. BPH monitoring is still strongly dependent on manmade investigation in rice fields and light trapping for the migratory populations (Hu et al., 2011; Liu, 2013; Masarudin et al., 2019). These methods have the obvious characteristics of low efficiency, high labor intensity, and high professional requirements. Although the image recognition and counting techniques (Yao et al., 2014; Yu et al., 2019) and spectral remote sensing (Huang et al., 2015; Ghobadifar et al., 2016a; Ghobadifar et al., 2016b; Liu and Sun, 2016) have been found to have potential for estimating the density of rice planthoppers, their practical application is still rare because these methods are restricted by environmental conditions. Therefore, a practical, simple, and automatic technique is demanded and promising in rice planthopper monitoring.

The chemical composition, such as chlorophyll, water, and soluble sugar, will change when plants are damaged by sap-sucking insects. Aphid infestation resulted in a significant reduction of chlorophyll content of crops (Lal, 1973; Burd and Elliott, 1996). Aphids *Lipaphis erysimi* (Kalt) and *Myzus persicae* (Sulzer) significantly reduced the nutritional constituents of mustard plants, such as lipid, carbohydrate, nitrogen, and protein concentrations (Pushpa and Sinhal, 2011). Infestation of the cotton-melon aphid *Aphis gossypii* induced a significant reduction of total soluble sugar contents in watermelon seedlings (Wu et al., 2015). The water potentials in young barley plants became lower after infestation with the green bug *Schizaphis graminum* (Cabrera et al., 1995). Rice planthopper damage also leads to changes in the chemical composition of rice. The infestation with brown planthopper *Nilaparvata lugens* decreased the contents of water, total chlorophyll, and free sugars of rice plants (Cagampang et al., 1974), and in the susceptible rice varieties, the chlorophyll contents in leaves decreased by more than 20% (Chen et al., 2003). However, the relationship between changes in the chemical composition of crops and the population density of pests damaging the crop is still vague. When this relationship unfolds, it will be useful for monitoring pest density.

**Figure 1:**
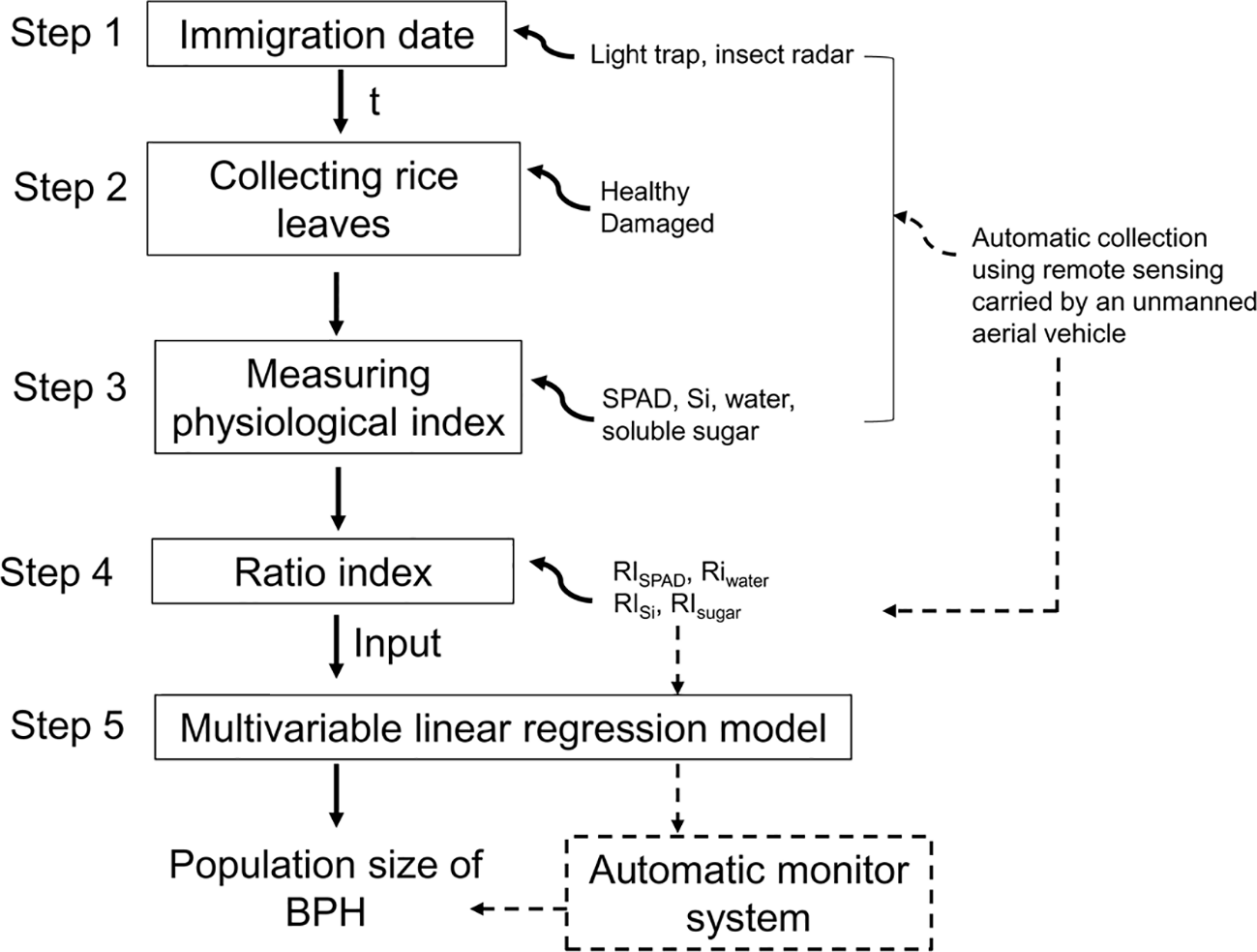
Flowchart of establishing a BPH monitor method based on the ratio physiological index of rice leaves. The solid line means the process has been established in this study, and the dashed line means it will be established. t means the damage duration of BPH.

So, in this study, we aimed to develop a new method for estimating BPH density based on physiological traits of rice plants and carried out studies on (1) the effect of BPHs on physiological traits of rice leaves, contents of chlorophyll (denoted using SPAD readings), water, soluble sugar, and silicon at different growth stages of rice plants; (2) establishing ratio physiological index (RPI) based on these physiological traits of the BPH-damaged and healthy rice leaves; (3) simulating the relationship between RPI and BPH density; and (4) establishing a general model based on RPI and testing the estimation accuracy in the following 2 years. A new nonprofessional and automatable method for estimating BPH density was developed by examining the physiological index of rice leaves (**Figure 1**).

## Materials and methods

### Insects and rice

The BPH, *Nilaparvata lugens*, was reared in the laboratory using rice seedlings at 25°C and 70%–80% RH. The rice cultivar was Wuyunjing 7 widely planted in Jiangsu province, China. The rice was planted in a plastic cup (diameter 8.4 cm, height 10.0 cm) with one hill of rice or in a pot (diameter, 33.0 cm; height, 32.5 cm) with three hills. Each hill of rice included three rice seedlings when transplanted.

### Effects of BPH on physiological traits of rice leaves

Two-day-old adults of BPH were released onto cup-grown rice plants. Seven population densities, 0, one, two, three, four, five, and six pairs of female and male adults per hill of rice, were designed to generate rice plants with different degrees of damage by BPHs. After the release of BPH, the rice plants were covered using a transparent plastic box to avoid the escape of BPH, and then the number of BPH was examined every day and new ones were added when the adults died to maintain a constant density of BPH on the rice plants. The release time of BPH adults was performed at four growth stages of rice: tillering, jointing, booting, and heading stages, respectively, to explore the effect of the rice growth stage on the density estimation of BPH. After 1 and 2 weeks (two damage durations), the upper three leaves of a rice plant were collected for examining the traits: SPAD readings and contents of water, Si, and soluble sugar. The experiment for each BPH density (seven densities in total) in a growth stage of rice plants was performed with four replicates. A combination of four BPH release times and two damage durations formed eight types of BPH-damaged cases. Each type of damage case included four replicates, and each replicate had seven sets of BPH densities. So, 8 × 4 × 7 = 224 cases of BPH damage were established in the cup-grown rice plants.

A chlorophyll meter (SPAD-502, Soil–Plant Analysis Development Section, Minolta Camera Co., Osaka, Japan) was used to examine the SPAD readings of a rice leaf. Three SPAD readings of a leaf were measured at three equal-part points, and the upper three leaves of a rice plant randomly collected in the cup-grown rice (Huang et al., 2015). The mean of all nine SPAD readings from a rice plant was considered the SPAD reading of a cup of rice. The relative water content of rice leaves was measured using the gravimetric method. The fresh weight (FW) of the upper three leaves of a rice plant was measured, and then these leaves were placed in a hot oven at 100°C to dry. The constant weight of the dried leaves was considered the dried weight (DW). The relative water content (H_2_O %) was computed using H_2_O % = (FW − DW)/FW × 100. The content of soluble sugar in rice leaves was measured using the anthrone-H_2_SO_4_ colorimetry method followed by Chang et al. (2022). The content of silicon in rice leaves was examined using an autoclave-induced digestion method (Elliott and Snyder, 1991). The rice leaves in all 224 cases of BPH damage were measured.

### Establishment of ratio physiological index of rice leaf and estimation models

The SPAD readings and contents of H_2_O, Si, and soluble sugar in rice leaves are affected not only by the BPH damage but also by the rice growth stage. To remove the effect of the latter, the ratio physiological index (RPI) of rice leaves was established using the ratio of a physiological trait value of BPH-damaged rice leaves to that of healthy rice leaves without BPHs. For example, the ratio physiological index of SPAD readings (RPI_SPAD_) was computed: RPI_SPAD_ = (SPAD readings of rice leaves damaged by BPH)/(SPAD readings of healthy rice leaves). Four ratio physiological indices, RPI_SPAD_, RPI_water_, RPI_Si_, and RPI_sugar_, based on the SPAD reading and contents of H_2_O, Si, and soluble sugar in leaves, were established, respectively.

Based on the RPI data collected from the cup-grown rice plants damaged by different BPH densities for 1 and 2 weeks mentioned above, the correlation between a PRI RPI_SPAD_, RPI_Si_, RPI_water_, or RPI_sugar_ and the BPH density damaging rice plants was analyzed using the Pearson method, and a significant correlation was found at almost all eight types of BPH-damaged case (**Table 1**). Therefore, we established eight multivariable linear regression models for estimating the BPH density (*N*_BPH_) in each type of damage case, respectively. For example, a model for estimating BPH density on the tillering rice damaged for 1 week was as follows: *N*_BPH-1_ = *C*_0_ + *C*_1_ × RPI_SPAD_ + *C*_2_ × RPI_Si_ + *C*_3_ × RPI_water_ + *C*_4_ × RPI_sugar_, where *C*_0_, *C*_1_, *C*_2_, *C*_3_, and *C*_4_ are constants. The *F* and *p*-values and the correlation coefficient between estimated and measured BPH density were used to assess the goodness-of-fit tests of a model. These eight case-specific models exhibited a good potential to estimate the population density of BPH, but their application range might be limited because they were rice growth stage and damage duration-specific.

**Table 1:** Coefficient of correlation between ratio physiological index of rice leaf and BPH density damaged rice for 7 and 14 days.

Growth stage of rice	Days after BPH release	Ratio physiological index of rice leaf			
		RPI_SPAD_	RPI_Si_	RPI_Water_	RPI_Sugar_
Tillering	7	−0.44631^*^	−0.51231^**^	−0.85476^**^	−0.67116^**^
	14	−0.79727^**^	−0.4940^**^	−0.86347^**^	−0.96312^**^
Jointing	7	−0.3026 ns	−0.30836 ns	−0.66492^**^	−0.83737^**^
	14	−0.79353^**^	−0.6824^**^	−0.79262^**^	−0.24186 ns
Booting	7	−0.69902^**^	−0.5012^**^	−0.33403 ns	−0.5545^**^
	14	−0.81396^**^	−0.85227^**^	−0.56798^**^	−0.16685 ns
Heading	7	−0.50377^**^	−0.83411^**^	−0.7385^**^	−0.79325^**^
	14	−0.69818^**^	−0.8020^**^	−0.77569^**^	−0.42107^*^

ns, not significant.

^*^p = 0.05; ^**^p = 0.01—levels of significance.

The RPI was derived from the ratio based on damaged to healthy rice leaves, so the value was almost independent of the rice growth stage. However, the damage duration (*t*) and population increase rate (*α*) of BPH will impact the RPI. So, a general RPI model including these two factors (*t* and *α*) was established here, *N*_BPH-_ *_t_* = *α* × (*t* − 1)(*C*_0_ + *C*_1_ × RPI_SPAD_ + *C*_2_ × RPI_Si_ + *C*_3_ × RPI_water_ + *C*_4_ × RPI_sugar_) (*t* ≥ 2), where RPI_SPAD_, RPI_Si_, RPI_water_, and RPI_sugar_ are the ratio physiological indices of SPAD readings and contents of Si, water, and soluble sugar in rice leaves measured at *t* weeks after BPH release or immigration. When *t* = 1, the model was *N*_BPH-1_ = *C*_0_ + *C*_1_ × RPI_SPAD_ + *C*_2_ × RPI_Si_ + *C*_3_ × RPI_water_ + *C*_4_ × RPI_sugar_. The increased rate of BPH (*α*) in a week can be empirically estimated via investigation. This general model can be used when the immigration peak of BPH has been known.

### Validity of the general model

We tested the validity of the general model in 2019 and 2020 using pot-grown rice plants with three hills of rice per pot. In the tillering stage, 0, one, two, three, four, five, and six pairs of female and male BPH adults were released into a pot of rice, respectively, and then the rice plants were covered using a transparent plastic box. The number of BPH on three hills of rice in a pot was investigated at 1, 3, 4, 5, 6, and 7 weeks after BPH release in 2019, and at 1, 3, 4, and 7 weeks in 2020. On the same day, the rice leaves were collected to examine the SPAD readings and contents of H_2_O, Si, and soluble sugar using the same method as mentioned above. Four and five replicates were performed for each population density in 2019 and 2020, originally released into a pot of rice plants, respectively. A total of 168 and 140 time-series samples were attained in 2019 and 2020, respectively. The increased rate of BPH in a week (*α*) was approximately 5/3 found in 2 years of investigation (*α* = 1.667). We used the general model mentioned above to evaluate the population size of BPH on pot-grown rice plants. The validity of the model was examined using a linear relationship between estimated and measured population sizes (*R* ^2^) and the root mean squared error (RMSE) based on 168 and 140 samples collected in 2019 and 2020, which were not used for establishing the model.

### Data analysis

Effects of BPH density and rice growth stage on these physiological traits: contents of chlorophyll (SPAD readings), H_2_O, Si, and soluble sugar of rice leaves were analyzed using the GLM model, and the data collected at 7 and 14 days after BPH release were considered repeat measurements. If the effect of BPH density or its interaction with the rice growth stage on a physiological trait of rice was significant, differences in this trait among BPH densities were analyzed using ANOVA followed by *post-hoc* Tukey’s test. A significant difference between healthy (no BPHs) and damaged (with BPHs) leaves indicated the potential of a physiological trait of leaves to monitor the BPH density. The correlation between RPI and BPH density, or between the estimated and measured BPH densities, was analyzed using the Pearson method. All the data analyses and simulations of the multivariable linear regression model were performed using IBM SPSS Statistics V25.

## Results

### Effect of BPH on SPAD readings of rice leaves

**Figure 2:**
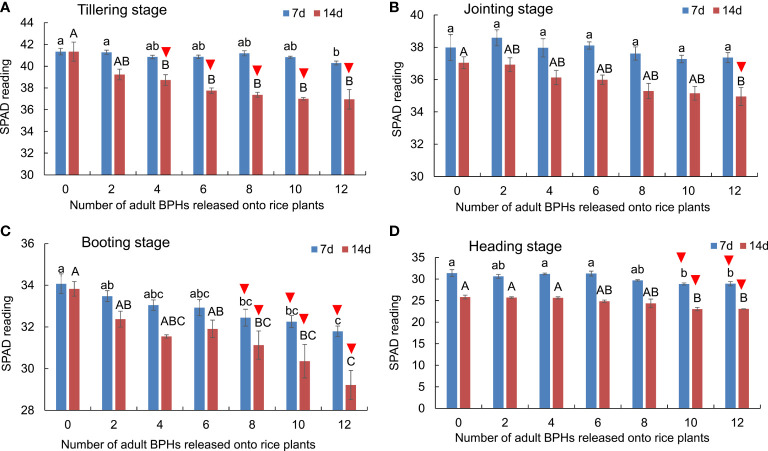
The SPAD readings of rice leaves at the tillering **(A)**, jointing **(B)**, booting **(C)**, and heading stages **(D)** were damaged by different numbers of BPHs for 7 and 14 days. The different lowercase and uppercase letters above the bar mean significant differences among different numbers of BPHs damaged for 7 and 14 days, respectively, analyzed by the *post-hoc* Tukey’s test. A triangle above the bar means those rice plants damaged by BPHs were distinguishable from the healthy rice based on the SPAD.

The BPH density (*F*_6, 84_ = 34.860, *p* < 0.001) and rice growth stage (*F*_3, 84_ = 1,998.145, *p* < 0.001) significantly affected the SPAD readings of rice leaves, and there was no significant interaction between them (*F*_18, 84_ = 1.556, *p* = 0.091). The SPAD readings decreased as rice plants were damaged by BPHs for 14 days at all four growth stages: the tillering (*F*_6, 21_ = 7.990, *p* < 0.001; **Figure 2A**), jointing (*F*_6, 21_ = 3.783, *p =* 0.01; **Figure 2B**), booting (*F*_6, 21_ = 7.425, *p* < 0.001; **Figure 2C**), and heading stages (*F*_6, 21_ = 6.701, *p* < 0.001; **Figure 2D**), and the SPAD reading also decreased at tillering (*F*_6, 21_ = 3.304, *p* = 0.019; **Figure 2A**), booting (*F*_6, 21_ = 5.076, *p* = 0.002; **Figure 2C**), and heading stages (*F*_6, 21_ = 5.632, *p* = 0.001; **Figure 2D**), except at the jointing stage (*F*_6, 21_ = 0.928, *p* = 0.495; **Figure 2B**) as BPH were damaged for 7 days. The SPAD readings could distinguish healthy rice plants without BPHs from damaged rice plants by four or more BPHs for 14 days at the tillering stage (**Figure 2A**), by 12 BPHs for 14 days at the jointing stage (**Figure 2B**), by eight or more BPHs for 7 or 14 days (**Figure 2C**), and by 12 or more BPHs for 7 days (**Figure 2D**).

### Effect of BPH on the content of silicon in rice leaves

**Figure 3:**
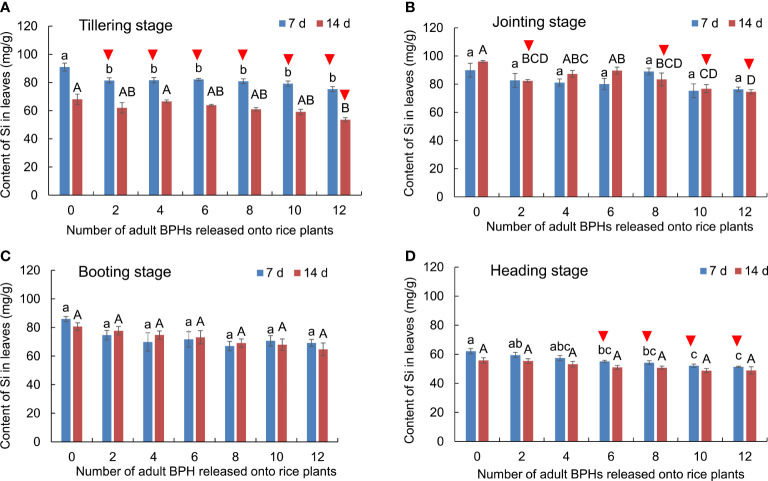
The content of Si in rice leaves at the tillering **(A)**, jointing **(B)**, booting **(C)**, and heading stages **(D)** damaged by 0–12 BPHs. The different lowercase and uppercase letters above the bar mean significant differences among different numbers of BPHs damaged for 7 and 14 days, respectively, analyzed by the *post-hoc* Tukey’s test. A triangle above the bar means those rice plants damaged by BPHs were distinguishable from healthy rice based on the Si content in leaves.

The BPH density (*F*_6, 84_ = 19.904, *p* < 0.001) and rice growth stage (*F*_6, 84_ = 228.993, *p* < 0.001) significantly affected the content of silicon (Si) in rice leaves, and there was no interaction between them (*F*_18, 84_ = 1.157, *p* = 0.316). BPH damage led to the decrease of Si content in rice leaves at the tillering (**Figure 3A**), jointing (**Figure 3B**), and heading stages (**Figure 3D**), whereas the damage did not affect the Si content of leaves at the booting stage (**Figure 3C**). At the tillering stage, the healthy and BPH-damaged rice could be distinguished by the Si content in leaves when BPHs were damaged for 7 days (*F*_6, 21_ = 6.221, *p* = 0.001; **Figure 3A**), and the healthy rice and 12 BPH-damaged rice could be distinguished when BPHs were damaged for 14 days (*F*_6, 21_ = 4.724, *p* = 0.003; **Figure 3A**). At the jointing stage, the healthy rice plants were distinguishable from the BPH-damaged rice by two, eight, 10, or 12 BPHs for 14 days using the Si content (*F*_6, 21_ = 8.605, *p* = 0.001; **Figure 3B**). At the heading stage, the healthy rice was distinguishable from the BPH-damaged rice based on Si content when they were damaged by six to 12 BPHs for 7 days (*F*_6, 21_ = 7.864, *p* < 0.001; **Figure 3D**).

### Effect of BPH on the content of water in rice leaves

**Figure 4:**
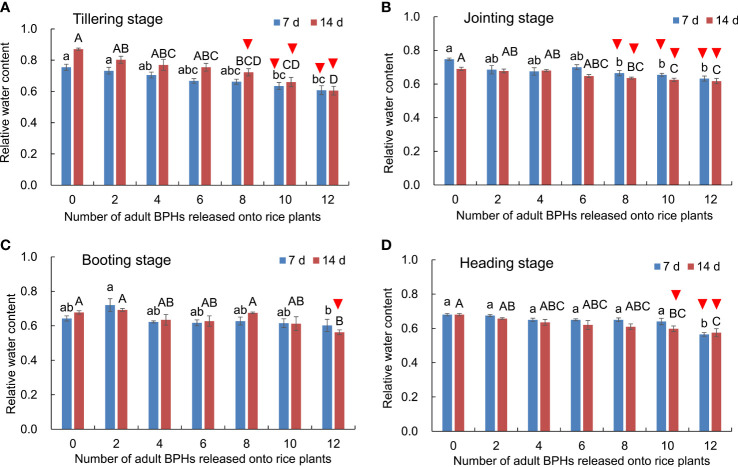
The content of water in rice leaves at the tillering **(A)**, jointing **(B)**, booting **(C)**, and heading stages **(D)** damaged by 0–12 BPHs. The different lowercase and uppercase letters above the bar mean significant differences among different numbers of BPHs damaged for 7 and 14 days, respectively, analyzed by the *post-hoc* Tukey’s test. A triangle above the bar means those rice plants damaged by BPHs were distinguishable from healthy rice based on the water content in leaves.

Effects of BPH density (*F*_6, 84 _= 41.606, *p* < 0.001), rice growth stage (*F*_3, 84 _= 51.339, *p* < 0.001), and their interaction (*F*_18, 84 _= 3.336, *p* < 0.001) on the content of water in rice leaves were significant (**Figure 4**). The water content in leaves decreased as the number of BPH-damaged rice plants increased at tillering (**Figure 4A**), jointing (**Figure 4B**), booting (**Figure 4C**), and heading stages (**Figure 4D**). At the tillering stage of rice, the healthy rice plants could be distinguished from the damaged ones by eight to 12 BPHs for 14 days based on the water content (*F*_6, 21 _= 11.895, *p* < 0.001; **Figure 4A**). At the jointing stage of rice, the healthy rice plants were distinguishable from damaged ones by eight to 12 BPHs for 7 days (*F*_6, 21 _= 4.906, *p* = 0.003; **Figure 4B**) and 14 days (*F*_6, 21 _= 8.272, *p* < 0.001; **Figure 4B**).

### Effect of BPH on the content of soluble sugar in rice leaves

**Figure 5:**
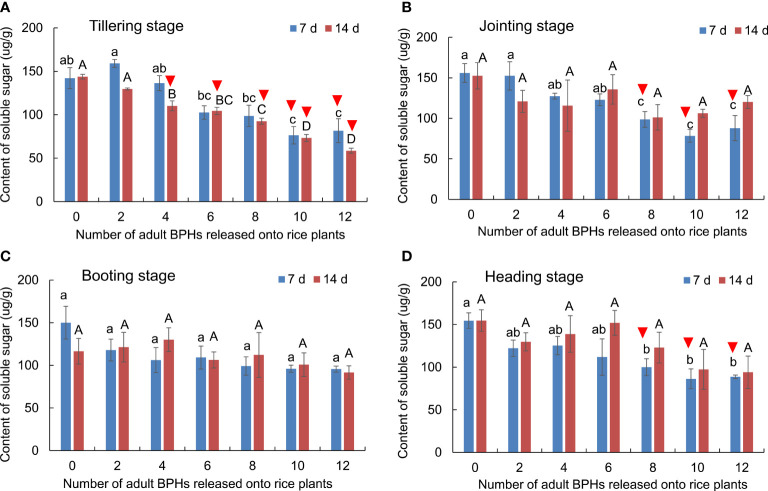
The content of soluble sugar in rice leaves at the tillering **(A)**, jointing **(B)**, booting **(C)**, and heading stages **(D)** damaged by different numbers of BPHs. The different lowercase and uppercase letters above the bar mean significant differences among different numbers of BPHs damaged for 7 and 14 days, respectively, analyzed by the *post-hoc* Tukey’s test. A triangle above the bar means those rice plants damaged by BPHs were distinguishable from healthy rice based on the content of soluble sugar in leaves.

The BPH density significantly affected the content of soluble sugar in rice leaves (*F*_6, 84_ = 17.983, *p* < 0.001), but the rice growth stage (*F*_3, 84 _= 2.601, *p* = 0.057) and its interaction with the BPH density (*F*_18, 84_ = 0.910, *p* = 0.569) did not significantly affect the content (**Figure 5**). The content of soluble sugar in rice leaves was significantly decreased by the damage of four to 12 BPHs for 14 days (*F*_6, 21 _= 9.636, *p* < 0.001) and by the damage of 10 and 12 BPHs for 7 days (*F*_6, 21 _= 8.272, *p* < 0.001) in the tillering stage of rice (**Figure 5A**). In the jointing (*F*_6, 21 _= 7.211, *p* < 0.001) and heading stages (*F*_6, 21 _= 4.064, *p* = 0.007), the eight to 12 BPH damage for 7 days significantly decreased the content of soluble sugar in leaves (**Figures 5B, D**). In the booting stage, the two to 12 BPH damage for 7 and 14 days did not affect the content of soluble sugar in leaves (**Figure 5C**). The rice damaged by BPHs could be distinguished using the content of soluble sugar in leaves at the tillering, jointing, and heading stages (**Figure 5**).

### Correlation between the ratio physiological index of rice leaf and population density of BPH

These four ratio physiological indices based on SPAD (RPI_SPAD_) and contents of Si (RPI_Si_), water (RPI_water_), and soluble sugar (RPI_sugar_) in leaves were significantly correlated with the density of BPHs damaging these rice plants for 7 and 14 days at four growth stages (**Table 1**). These ratio indices, derived from rice leaves, exhibited great potential to estimate the population density of BPH.

### Models for monitoring population density of BPH

The multivariable linear regression models based on these four ratio physiological indices, RPI_SPAD_, RPI_Si_, RPI_water_, and RPI_sugar_ of rice leaves, were established for estimating the BPH density on the tillering, jointing, booting, and heading stages of rice damaged for 1 week (*N*_BPH-1_) and 2 weeks (*N*_BPH-2_). There were significant correlations between the measured and estimated population density based on these models (**Table 2**). To extend the applied range to all growth stages of rice, a general model was set up to estimate the population density of BPH (*N*_BPH-_ *_t_*) damaging rice for *t* weeks (*t* ≥ 2), *N*_BPH-_ *_t_* = 1.667 × (*t* − 1)(71.91807 − 31.4125 × RPI_SPAD_ − 15.6221 × RPI_Si_ − 16.9861 × RPI_water_ − 7.34955 × RPI_Sugar_) based on these four ratio physiological indices of rice leaves.

**Table 2:** Models for estimating the density of BPH on four growth stages of rice plants damaged for 1 and 2 weeks based on four ratio physiological indices of rice leaf and the coefficient of correlation between measured and estimated BPH density using the model (*R* ^2^).

Growth stage	Week	Model	*F*	*p*-value	*R* ^2^
Tillering	1	*N*_BPH-1 = _94.288 − 43.468 × RPI_SPAD_ − 20.522 × RPI_Si_ − 24.466 × RPI_water_ − 5.672 × RPI_sugar_	82.265	<0.001	0.9668^**^
	2	*N*_BPH-2 = _27.340 − 8.542 × RPI_SPAD_ − 2.925 × RPI_Si_ + 2.673 × RPI_water_ − 18.405 × RPI_Sugar_	105.287	<0.001	0.9696^**^
Jointing	1	*N*_BPH-1 = _48.283 − 12.898 × RPI_SPAD_ − 5.042 × RPI_Si_ − 16.012 × RPI_water_ − 13.506 × RPI_Sugar_	25.640	<0.001	0.9038^**^
	2	*N*_BPH-2 = _106.861 − 54.090 × RPI_SPAD_ − 10.881 × RPI_Si_ − 38.511 × RPI_water_ − 2.807 × RPI_Sugar_	31.026	<0.001	0.9185^**^
Booting	1	*N*_BPH-1 = _97.392 − 76.842 × RPI_SPAD_ − 3.516 × RPI_Si_ − 10.606 × RPI_water_ − 4.982 × RPI_Sugar_	10.425	<0.001	0.8028^**^
	2	*N*_BPH-2 = _64.468 − 30.831 × RPI_SPAD_ − 28.889 × RPI_Si_ − 2.022 × RPI_water_ − 1.885 × RPI_Sugar_	27.295	<0.001	0.9088^**^
Heading	1	*N*_BPH-1 = _58.865 − 6.219 × RPI_SPAD_ − 23.797 × RPI_Si_ − 18.550 × RPI_water_ − 10.637 × RPI_Sugar_	92.652	<0.001	0.9703^**^
	2	*N*_BPH-2 = _77.847 − 18.411 × RPI_SPAD_ − 29.404 × RPI_Si_ − 28.397 × RPI_water_ − 0.901 × RPI_Sugar_	49.435	<0.001	0.9465^**^

^**^ p < 0.01—level of significance.

### Validation of the general model

**Figure 6:**
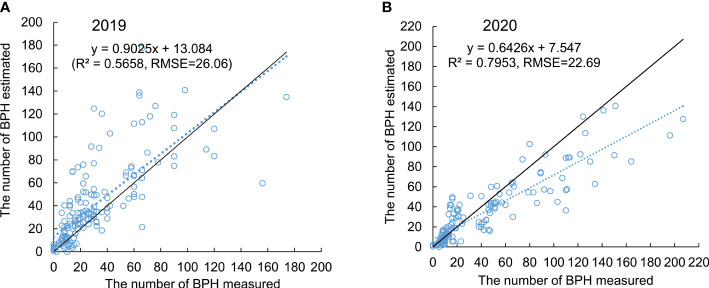
Validity of the general model tested in 2019 **(A)** and 2020 **(B)**. The dashed line presents the relationship between the measured (x) and estimated (y) density of BPH using the general model. The solid line presents x = y.

The validation of the general model was tested in 2 years, 2019 and 2020, using the pot-grown rice plants. The model exhibited higher accuracy when estimating the population density of BPH in both 2019 (**Figure 6A**) and 2020 (**Figure 6B**) after 1–7 weeks from release. The *R* ^2^ values between the measured and estimated density of BPH on three hills of rice were 0.5658 and 0.7953 in 2019 (*n* = 168) and 2020 (*n* = 140), respectively, and the RMSEs for the predication were 26.06 and 22.69, respectively, indicating a good linear relationship (**Figure 6**). The general model based on four ratio physiological indices of rice leaves exhibited the potential for estimating BPH density on rice plants.

## Discussion

Rice planthopper damage causes changes in the chemical composition of rice leaves (Cagampang et al., 1974; Chen et al., 2003). In this study, we found that the contents of chlorophyll (SPAD readings), water, silicon, and soluble sugar in rice leaves were reduced as the BPH damage degree increased. Previous studies showed that SPAD readings, which are an indicator of the content of chlorophyll in rice leaves, were significantly decreased due to BPH infestation (Watanabe and Kitagawa, 2000; Huang et al., 2015). The SPAD readings of wheat were also reduced as they were damaged by aphids (Franzen et al., 2008). BPH damage decreased the number of silica cells in rice leaf sheaths (Yang et al., 2017) and the contents of water and free sugars in rice plants (Cagampang et al., 1974). These four physiological traits, SPAD, water, Si, and soluble sugar in rice leaves, exhibited similar responses to BPH damage, suggesting great potential to estimate the population density of BPH on rice plants.

The four physiological traits, SPAD readings, and contents of water, Si, and soluble sugar in rice leaves were significantly related to the damage caused by BPH at the tillering, jointing, booting, and heading stages of rice. This result suggests that these four traits might be used to establish a model for monitoring BPH. To reduce the effect of different growth stages of rice on physiological traits, we established the ratio physiological index based on BPH-damaged and healthy rice. The four ratio physiological indices of rice leaves were negatively correlated with the density of BPH damaging the rice plants, although one or two indices were not significant in a specific damage case. Therefore, the accuracy of a model based on a single physiological index of rice leaf to monitor BPH may be lower. So, the multivariable linear regression models based on four ratio physiological indices were established in this study, which did exhibit high goodness of fit and estimated accuracy. Most prediction models for rice planthopper were weather-based, and these weather factors, temperature, humidity, and rainfall, were often used (Prasannakumar and Subhash, 2014; Chen et al., 2015; Srinivasa et al., 2020). However, factors derived from rice plants were rarely used for monitoring rice planthoppers. To our knowledge, this is the first time to develop the ratio physiological indices of rice leaf to estimate BPH density.

The general model developed in this study using ratio physiological indices of rice leaf to estimate BPH density has the advantages of convenient utilization, good estimating accuracy, and a broad prospect of application. Leaf collection in rice fields is more convenient than surveying samples of BPH. Sampling for BPHs is highly professional and labor-intensive (Wang et al., 2014; Masarudin et al., 2019). Collecting rice leaves can be carried out by nonprofessionals, and this job is relatively easier than sampling BPHs. The accuracy was 30%–70% depending on the population density of BPHs and the growth stage of rice in the survey based on the commonly used method of shake or beat sampling (Qi et al., 1995). Although a gap remained between the measured and estimated density of BPHs using the general model established in this study, the overall trend of all estimated values was in good agreement with the practical cases (**Figure 6**), suggesting reasonable accuracy. Moreover, this general model was established based on the ratio physiological indices between the healthy and damaged rice plants at four growth stages, and two crucial factors, damage duration (*t*) and population increase rate of BPH (*α*), which related to changes in rice leaves in physiological traits, were considered, suggesting that the application of this general model may be suitable to almost all damage cases of rice when the immigration peak is clear. The examination of the physiological traits of rice leaf can be performed using the standard methods in the laboratory, and this examination will be performed automatically on an assembly line in the laboratory. So, this method based on the physiological traits of rice leaves may automate the estimation of BPH density and have a broad prospect of application.

Based on this study, the BPH density estimation may be performed via five simple steps, (1) determination of BPH immigration peak using light trapping or radar observation methods (Riley et al., 1994; Qi et al., 2010) to attain the damage duration (*t*, week) of BPH populations; (2) collection of rice leaves in rice fields, especially the healthy leaf without BPH damage; (3) examination of SPAD and contents of water, Si, and soluble sugar in sampled rice leaves in the laboratory; (4) establishment of ratio physiological indices based on the heathy and BPH-damaged leaves; and (5) estimating the population density of BPH based on the general model (**Figure 1**). Collected rice leaves need to be retained freshly before examination. An ice box can be used when sampling. The healthy rice leaves can be determined via a few visual scouts or by using the leaves with the lowest physiological traits in all collected leaves. So, the sampling of rice leaves is simple.

Hyperspectral remote sensing is a nondestructive method to measure the chemical composition of plants. The nitrogen nutritional status and chlorophyll content of rice plants can be diagnosed using hyperspectral data (Tan et al., 2008; An et al., 2020). Hyperspectral imaging can measure the contents of sugar and water in plants (Guo et al., 2007; Kim et al., 2015). The digital images can be collected using unmanned aerial vehicles (Roosjen et al., 2020). When these four physiological traits of rice leaves, SPAD, water, Si, and soluble sugar, are measured via hyperspectral remote sensing, BPH monitoring will be accomplished automatically in rice fields using a sampling–monitoring system carried by an unmanned aerial vehicle (**Figure 1**). Therefore, an automatic and intelligent method based on the physiological traits of rice leaves will be developed for monitoring BPH density, which greatly enhances working efficiency and estimation accuracy.

## Conclusion

The physiological traits of rice leaves are strongly correlated with the number of brown planthoppers damaging rice plants. The ratio physiological index of rice leaf damaged by brown planthoppers to healthy rice is effective in monitoring the population density of brown planthoppers based on the SPAD reading and contents of water, silicon, and soluble sugar in the leaf. The regression model based on four ratio physiological indices of rice leaf, the damage duration, and the population increase rate exhibits high goodness of fit and estimation accuracy. The new method based on the ratio physiological index of crops will promote the development of pest density estimation toward nonprofessionalization and automation.

## Data availability statement

The original contributions presented in the study are included in the article/supplementary material. Further inquiries can be directed to the corresponding author.

## Author contributions

X-DL and MC conceived the ideas and designed methodology. MC collected the data. X-DL and MC analyzed the data. X-DL led the writing of the manuscript. All authors contributed the article and approved the submitted version.
